# Supplementary material for: The NDV-3A vaccine protects mice from multidrug resistant Candida auris infection
Source: PLoS Pathog. 2019 Aug 5;15(8):e1007460. doi: 10.1371/journal.ppat.1007460 (PMC6695204; doi:10.1371/journal.ppat.1007460)
Supplement: S1 Dataset — (A) Comparison between C. albicans Als3p (GenBank: AOW31402.1) and its homolog on C. auris (GenBank: PIS50650.1) using CLUSTAL-W. (B) Comparison between C. albicans Als3p (GenBank: AOW31402.1) and its homolog on C. auris (GenBank: PIS50263.1) using CLUSTAL-W. (C) Comparison between C. albicans Als3p (GenBank: AOW31402.1) and its homolog on C. auris (GenBank: XP_018167572.2) using CLUSTAL-W. (DOCX) [file ppat.1007460.s001.docx]

**S1 Dataset:** **Protein sequence alignment. (A)** Comparison between *C. albicans* Als3p (GenBank: AOW31402.1) and its homolog on *C. auris* (GenBank: PIS50650.1) using CLUSTAL-W.

AOW31402.1 1 -MLQQYTLLLIYLSVATAKTITGVFNSFNSLTWSNAATYNYKGPGTPTWNAVLGWSLDGT
PIS50650.1 1 MKLASLAAVSLAVAGAVAKLQSGVFNGIKSITPS-----DNRRPEQPSWHATVSWEIKPA

AOW31402.1 60 S-ASPGDTFTLNMPCVFKFTTSQTSVDLTAHGVKYATCQFQAGEEFMTFSTLTCTVSNTL
PIS50650.1 56 MGVQEGDTFTLHMPYVYKFTSSSNTLQLTAGGQVVANCNLYSGENIVGYSEVQCTATAAA

AOW31402.1 119 TPSIKALGTVTLPLAFNVGGTGSSVDLEDSKCFTAGTNTVTFNDGGKKISINVDFER---
PIS50650.1 116 ANAGTFTGDVTFPFTFNAGSTSDEVNLEAAGVWKSGQNTVTWSDGDKSFSTTVDFNPGAS

AOW31402.1 176 --SNVDPKGYLTDSRVIPSLNKVSTLFVAPQCANGYTSGTMGFANTYGDVQIDCSNIHVG
PIS50650.1 176 SIIQGSPENGVYGLRKMVSLNINQHYLMGPSCPYDGQYGRLEISNPSPGVGFDCSSLAGA

AOW31402.1 234 ITKGLNDWNYPVSSESFSY-TKTCSSNGIFITYKNVPAGYRPFVDAYISATDVNSYTLSY
PIS50650.1 236 ITDQVNDWYFPKTAEKIGVNIDSCSSYQATVSFSNLPAGFRPYININAAIPNVASFRSSN

AOW31402.1 293 ANEY-TCAGGYWQRAPFTLRWTGYRNSDAGSNG---------------------------
PIS50650.1 296 TYSYNFVCGGRRQSGQSSIAWVMYNNGNTGSGGDFKPVVVTTVTDPDITTTAVVTSTGTS

AOW31402.1 325 -----IVIVATTRTVTDSTTAVTTLPFDPNRDKTKTIEILKPIPTTTITTSYVGVTTSYS
PIS50650.1 356 TNTIVVSVPISVSTVTHTGTDSVATTHTTTKSGTRIISIDVPTPTTTITSTWTGSTTETI

AOW31402.1 380 TKTAPIGETATVIVDIPYHTTTTVTSKWTGTITSTTTHTNPTDSIDTVIVQVPSPNPTVT
PIS50650.1 416 TVPASSSGGTNTVVVEVPTPTTTVTRTWTGTVTSTEIIPAPSGGTATVIVDVPTP--VTT

AOW31402.1 440 TTEYWSQSFATTTTITGPPGNTDTVLIREPPNHTVTTTEYWSESYTTTSTFTAPPGGTDS
PIS50650.1 474 VTRTWTGSVTSTETIPAKPGGTETVVIDVPTPSTTIYSTWTGTETTTRTIPASSPGGTDT

AOW31402.1 500 VIIKEP---------------------------------PNPTVTTTEYWSESYTTTTTV
PIS50650.1 534 VFIEVPTPVTVITKTWTGSYTTVVTEPFVSGTQTIVVEVPTPTTTITRTWTGTTSQTETL

AOW31402.1 527 TAPPGGTDTVIIREPPNHTVTTTEYWSQSYTTTTTVIAPPGGTDSVIIREPPNPTVTTTE
PIS50650.1 594 PAPSGGTQTVVVDVP-TPYTTITKTWTGSVSTTETQPAPSGGTGTVIVEVP-TPVTTITR

AOW31402.1 587 YWSQSYATTTTITAPPGETDTVLIREPPNHTVTTTEYWSQSYATTTTITAPPGETDTVLI
PIS50650.1 652 TWTGTTTQTETIPAPSGGTETIIVDVP-TPVTTVTSTYTGSITTTRTIPAPSGGTETVVV

AOW31402.1 647 REP-----------------------------------PNHTVTTTEYWSQSYTTTTTVI
PIS50650.1 711 EVPTPTTTIYSTWTGTVTSTRTDHATTSGGTDTLVIEVPTPVTTITKTWTGSITTTETIP

AOW31402.1 672 APPGGTDSVIIKEPPNPTVTTTEYWSQSYATTTTITAPPGETDTVLIREPPNHTVTTTEY
PIS50650.1 771 APSGGTETVIVEVP-TPVTTITRTWTGSVTTTETLTAPSGGTETVIIDVP-TPVTTITRT

AOW31402.1 732 WSQSYATTTTITAPPGETDTVLIREPPNHTVTTTEYWSQSFATTTTVTAPPGGTDTVIIR
PIS50650.1 829 WTGTTTQTETLPAPSGGTETIIIDVP-TPVTTITRTWTGSVTSTETLTAPSGGTETVIID

AOW31402.1 792 EPPNHTVTTTEYWSQSFATTTTIIAPPGETDTVLIREPPNPTVTTTEYWSQSYTTATTVT
PIS50650.1 888 VP-TPVTTITKTWTGSVTTTETIPAPSGGTETVIIDVP-TPVTTFTRTWTGTSTQTETIP

AOW31402.1 852 APPGGTDTVIIYDTMSSSEISSFSRPHYTNHTTLWSTTWVIETKTITETSCEGDKGCSWV
PIS50650.1 946 APFGGTETVIIDVPTPVTTITRTWTGSTTTTETIPAETGGTETVIIDVPTPVTTVTKTWT

AOW31402.1 912 SVSTRIVTIPNN--------IETPMVTNTVDTTTTESTLQSPSGIFSESG----VSVETE
PIS50650.1 1006 GSITTTETIPAQSGGTETVVVEVPTPTTTIWSTWTGTETATRTVPASTSGGTDTVIVEVP

AOW31402.1 960 SSTFTTAQTNPSVPTTESEVVFTTKGNNGNGPYESPSTNVKSSMDENSEFTTSTAASTST
PIS50650.1 1066 TPVTVITKTWTGSYTTVVTEPFESGTQTIVVEVPTPTTTVTSTWTETTTAVTTIPATSSG

12/14, 85 %

15/17, 88 %

14/17, 82 %

15/18, 83 %

12/16, 75 %

13/15, 87 %

12/14, 85 %

13/19, 68 %

17/22, 77 %

13/20, 65 %

22/27, 81 %

AOW31402.1 1020 DIENETIATTGSVEASSPIISSSADETTTVTTTAESTSVIEQQTNNNGGGNAPSATSTSS
PIS50650.1 1126 GTDTVVVQVPTPMTTITKTWTGTVTTTETLPTVSGETVTVVVDVPSQYTTLTSTWTGTST

AOW31402.1 1080 ---------PSTTTTANSDSVITSTTSTNQSQSQSNSDTQQTTLSQQMTSSLVSLHMLTT
PIS50650.1 1186 RTITEPPSGSDTVGTVVVEVPPTSTAYTTLTSTWTGSTTRIFTEPPAGSDTVGTIVVEVP

AOW31402.1 1131 FDGSGSVIQHSTWLCGLITLLSLFI---
PIS50650.1 1246 QTSTEYSTVTSTWTGSVTTTVTHPPTGS

**(B)** Comparison between *C. albicans* Als3p (GenBank: AOW31402.1) and its homolog on *C. auris* (GenBank: PIS50263.1) using CLUSTAL-W.

10/15, 66 %

AOW31402.1 1 MLQQYTLLLIYLSVATAKTITGVFNSFNSLTWSNAATYNYKGPGTPTWNAVLGWSLDG-T
PIS50263.1 1 ---MVLASFLLGLAACSASVNASVKEGIFDKILSITPPEDSLPETPSWSATVEWSFDEKN

AOW31402.1 60 SASPGDTFTLNMPCVFKFTTSQTSVDLTAHGVKYATCQFQAGEEFMTFSTLTCTVSNTLT
PIS50263.1 58 GVKAGDTFVLHMPYVYKFTSGTRAVDLVADSVTFANCDLFSGDNVVAYSELKCTATSACE

AOW31402.1 120 PSIKALGTVTLPLAFNVGGTGSSVDLEDSKCFTAGTNTVTFNDGGKKISINVDFERSNVD
PIS50263.1 118 KVNSAKGTVEFPFTFNAGSSSDKANLEASTVWHAGSNVVKWTDGSKELHSEVYFDEGNPY

AOW31402.1 180 PKGYLTDS-----RVIPSLNKVSTLFVAPQCANGYTSGTMGFANTYGDVQIDCSNIHVGI
PIS50263.1 178 LFSGSVEHGVYWLRKAVMRNTNQHLVLGPSCDCDGMSGYIEIQNPDYGVELDCSSVVGTI

AOW31402.1 235 TKGLNDWNYPVSSE-SFSYTKTCSSNGIFITYKNVPAGYRPFVDAYISATDVNSYTLSYA
PIS50263.1 238 TKQINDFYFPESAESCSVHVDECCATRVRVSFDNIPSGFRPYINVDSAIPHYDFNSRNQY

AOW31402.1 294 NEYTCAGGYWQRAPFTLRWTGYRNSDAGSNGIVIVATTRTVTDSTTAVTTLPFDPNRDKT
PIS50263.1 298 SYGFSCGGIELCDSLWTNWYMYKDGETGGDGEFNSIVVTTVTDSS---------------

AOW31402.1 354 KTIEILKPIPTTTITTSYVGVTTSYSTKTAPIGETATVIVDIPYHTTTTVTSKWTGTITS
PIS50263.1 343 -------------------ASTTGLTTITGPSTNTIVVTVPG------------------

AOW31402.1 414 TTTHTNPTDSIDTVIVQVPSPNPTVTTTEYWSQSFATTTTITGPPGNTDTVLIREPPNHT
PIS50263.1 366 ----------------ITPSETSSYDITSSWETSFSETTSWE------ESSYSQSTDSVI

AOW31402.1 474 VTTTEYWSESYTTTSTFTAPPGGTDSVIIKEPPNPTVTTTEYWSESYTTTTTVTAPPGGT
PIS50263.1 404 ESEHSSWSEYSSTSEYSSS-----------------------WSEHFSSYTGYNS-----

AOW31402.1 534 DTVIIREPPNHTVTTTEYWSQSYTTTTTVIAPPGGTDSVIIREPPNPTVTTTEYWSQSYA
PIS50263.1 436 ---------SFSVGTSTSKTQSLPTTLVIGSTTESIISSTTVSDTTTFVSSSTGFSESTG

AOW31402.1 594 TTTTITAPPGETDTVLIREPPNHTVTTTEYWSQSYATTTTITAPPGETDTVLIREPPNHT
PIS50263.1 487 HSSSNSKSIPEVPTNYPSVSSRVSTGVSTGVSTGVSTGVSTGVSTGVSTGIFTRASSDLS

AOW31402.1 654 VTTTEYWSQSYTTTTTVIAPPGGTDSVIIKEPPNPTVTTTEYWSQSYATTTTITAPPGET
PIS50263.1 547 SSISSDLTE---------------------------------------------------

AOW31402.1 714 DTVLIREPPNHTVTTTEYWSQSYATTTTITAPPGETDTVLIREPPNHTVTTTEYWSQSFA
PIS50263.1 556 ------SPSTFYTTVTSTWTNSITSTETHTAPSKDTVTVVVEVPSESVPCTTITSTWTGT

AOW31402.1 774 TTTTVTAPPGGTDTVIIREPPNHTVTTTEYWSQSFATTTTIIAPPGETDTVLIREPPNPT
PIS50263.1 610 TTAYTTVCVSDETTVIVQVPTTTTTTVEESG----AGHTTPHVPESTGQTLKVPVTSSST

AOW31402.1 834 VTTTEYWSQSYTTATTVTAPPGGTDTVIIYDTMSSSEISSFSRPHYTNHTTLWSTTWVIE
PIS50263.1 666 IVASHDESTNGGSDRTLTAVTSSTTIVNVPQSIRSNENLHSSNPGAPQQPAASTESGAEH

AOW31402.1 894 TKTITETSCEGDKGCSWVSVSTRIVTIPNNIETPMVTNTVDTTTTESTLQSPSGIFSESG
PIS50263.1 726 HKTLNPNQPEGGN-----------------------------------------------

AOW31402.1 954 VSVETESSTFTTAQTNPSVPTTESEVVFTTKGNNGNGPYESPSTNVKSSMDENSEFTTST
PIS50263.1 739 ------------------------------------------------------------

AOW31402.1 1014 AASTSTDIENETIATTGSVEASSPIISSSADETTTVTTTAESTSVIEQQTNNNGGGNAPS
PIS50263.1 739 ---------NDSTYVVHGGKESSTTVAEGVNNPTPSNVEYPSASSLVQSPGNDHLTASPS

AOW31402.1 1074 ATSTSSPSTTTTANSDSVITSTTSTNQSQSQSNSDTQQTTLSQQMTSSLVSLHMLTTFDG
PIS50263.1 790 LFTTSAVQVASSSSPASYVVSTYEGLAAGSTAS---------------------------

9/12, 75%

12/14, 86 %

16/24, 66 %

13/16, 81 %

AOW31402.1 1134 SGSVIQHSTWLCGLITLLSLFI
PIS50263.1 823 ---------FSVVLLTIFYLFV

**(C)** Comparison between *C. albicans* Als3p (GenBank: AOW31402.1) and its homolog on *C. auris* (GenBank: XP_018167572.2) using CLUSTAL-W.

8/11, 72 %

AOW31402.1 1 --MLQQYTLLLIYLSVATAKTITGVFNSFNSLTWSNAATYNYKGPGTPTWNAVLGWSLDG
XP_018167572.2 1 MKIISLLWCLALLYGNALAAPQTGVFTSIDSLTP-----FDVAWPMMPGWDATVSWHINS

AOW31402.1 59 -TSASPGDTFTLNMPCVFKFTTSQTSVDLTAHGVKYATCQFQAGEEFMTFSTLTCTVSNT
XP_018167572.2 56 SMEMKDGDTFFLRIPFVIEFNTDESSIQMSDGTNTFANCVLTPGENLVPYSEVKCTATTQ

AOW31402.1 118 LTPSIKALGTVTLPLAFNVGGTGSSVDLEDSKCFTAGTNTVTFNDGGKKISINVDFE---
XP_018167572.2 116 VEDVQSSSGTITFPIVFNAGFSAQESDLKAANHWRTGSNTLEWTDGSNTLTHPITFVGGT

AOW31402.1 175 --RSNVDPKGYLTDSRVIPSLNKVSTLFVAPQCANGYTSGTMGFANTYGDVQIDCSNIHV
XP_018167572.2 176 MSAFNGRPKRGILDQRSFVSTNTIRQFLMGPSCHSSDMSGELSIENLSEEAPFDCDSITT

AOW31402.1 233 GITKGLNDWNYPVSS-ESFSYTKTCSSNGIFITYKNVPAGYRPFVDAYIS-ATDVNSYTL
XP_018167572.2 236 AMSNQINAWYFPQTADEAEATIVSCSAAGVNVAFSNLPAGFRPYINIDATKKIAVSEIDN

AOW31402.1 291 SYANEYTCAGGYWQRAPFTLRWTGYRNSDAGSNGIVIVATTRTVTDSTTAVTTLPFDPNR
XP_018167572.2 296 IYHYNFTCNGAELSDSIFAA-WDQFFSDDTEEDDTLTQVVVTTATD--------------

AOW31402.1 351 DKTKTIEILKPIPTTTITTSYVGVTTSYSTKTAPIGETATVIVDIPYHTTTTVTSKWTGT
XP_018167572.2 341 ----------PGITATSIATHTGTDANTIVVNVPISESTVIVTGTNSIATTKTFTSSG--

AOW31402.1 411 ITSTTTHTNPTDSIDTVIVQVPSPNPTVTTTEYWSQSFATTTTITGPPGNTDTVLIREPP
XP_018167572.2 389 ---------------TRIVSIDTPIPTSTITSTWTESVTSTYTVPASPG-----------

AOW31402.1 471 NHTVTTTEYWSESYTTTSTFTAPPGGTDSVIIKEPPNPTVTTTEYWSESYTTTTTVTAPP
XP_018167572.2 423 --------------------------VTASVIVEVPIPTTTITQTWTGMETMTQTLPAEI

AOW31402.1 531 GGTDTVIIREPPNHTVTTTEYWSQSYTTTTTVIAPPGGTDSVIIREPPNPTVTTTEYWSQ
XP_018167572.2 457 GETQSVIVNIPDSTTIMSSNEATSEQTMSSSVDTIP------------------------

AOW31402.1 591 SYATTTTITAPPGETDTVLIREPPNHTVTTTEYWSQSYATTTTITAPPGETDTVLIREPP
XP_018167572.2 493 --TSSMNSSTSSGASLSEEISTQETSLSSQSSDESVSQTESYTASDPSPPVSSSFLNTSS

AOW31402.1 651 NHTVTTTEYWSQSYTTTTTVIAPPGGTDSVIIKEPPNPTVTTTEYWSQSYATTTTITAPP
XP_018167572.2 551 SKIGSQSLLSSQSDPVSTSEMAPQSGISSYITS------------WIGSITVPSSLSAET

AOW31402.1 711 GETDTVLIREPPNHTVTTTEYWSQSYATTTTITAPPGETDTVLIREPPNHTVTTTEYWSQ
XP_018167572.2 599 DS-----AQETSYHTTKSPTDNSASENVYSTESKKTENTDTKAS-----HSVISTSGTSD

AOW31402.1 771 SFATTTTVTAPPGGTDTVIIREPPNHTVTTTEYWSQSFATTTTIIAPPGETDTVLIREPP
XP_018167572.2 649 INDSSSDAYIDPSISTTTIG----------------------------------------

AOW31402.1 831 NPTVTTTEYWSQSYTTATTVTAPPGGTDTVIIYDTMSSSEISSFSRPHYTNHTTLWSTTW
XP_018167572.2 669 ------------------------------------------------VPSISVNWNSSF

AOW31402.1 891 VIETKTITETSCEGDKGCSWVSVSTRIVTIPNNIETPMVTNTVDTTTTESTLQSPSGIFS
XP_018167572.2 681 VITTLVS---------------------------ETTCISSTPKTVSETSDIQDLFSTCK

AOW31402.1 951 ESGVSVETESSTFTTAQTNPSVPTTESEVVFTTKGNNGNGPYESPSTNVKSSMDENSEFT
XP_018167572.2 714 EASAKTNNEGSLKASKLS-KSTSKIDTKVIFPTS---------------------EPDFI

AOW31402.1 1011 TSTAASTSTDIENETIATTGSVEASSPIISSSADETTTVTTTAESTSVIEQQTNNNGGGN
XP_018167572.2 752 ASTSSYWSQDIQ----SSNQFNIMTSPDISSAQSGPTELASARCTSANCGTQTNSKG---

AOW31402.1 1071 APSATSTSSPSTTTTANSDSVITSTTSTNQSQSQSNSDTQQTTLSQQMTSSLVSLHMLTT
XP_018167572.2 805 ---QNPNVATSLMSISNSDAPAVSPSSSQ-------------------------LPSVLT

8/12, 67 %

9/14, 64 %

17/22, 77 %

11/15, 73 %

8/10, 80 %

8/11, 72 %
