# Supplementary figures and images for: The NDV-3A vaccine protects mice from multidrug resistant Candida auris infection
Source: PLoS Pathog. 2019 Aug 5;15(8):e1007460. doi: 10.1371/journal.ppat.1007460 (PMC6695204; doi:10.1371/journal.ppat.1007460)

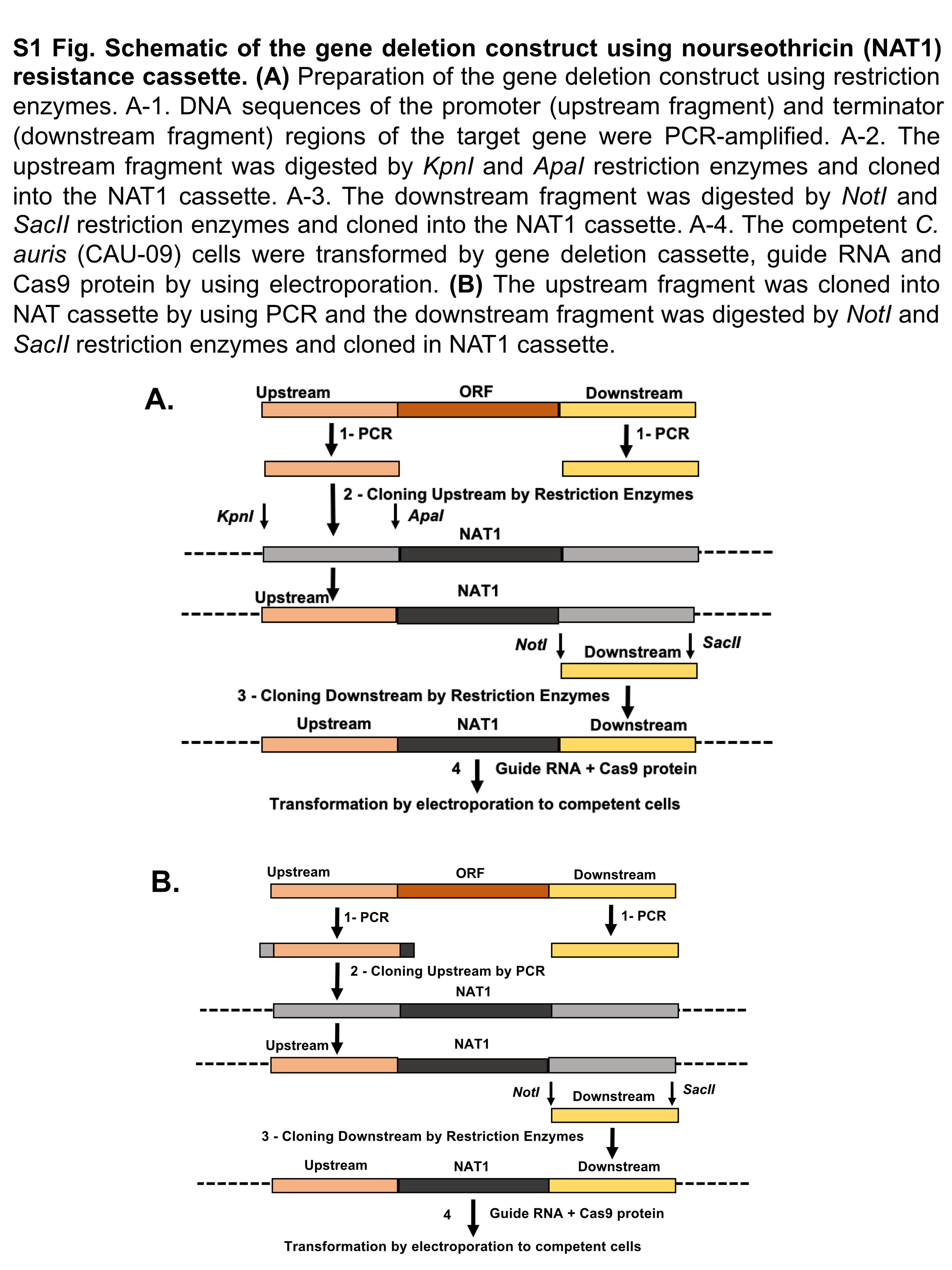

Supplement: S1 Fig — (A) Preparation of the gene deletion construct using restriction enzymes. A-1. DNA sequences of the promoter (upstream fragment) and terminator (downstream fragment) regions of the target gene were PCR-amplified. A-2. The upstream fragment was digested by KpnI and ApaI restriction enzymes and cloned into the NAT1 cassette. A-3. The downstream fragment was digested by NotI and SacII restriction enzymes and cloned into the NAT1 cassette. A-4. The competent C. auris (CAU-09) cells were transformed by gene deletion cassette, guide RNA and Cas9 protein by using electroporation. (B) The upstream fragment was cloned into NAT cassette by using PCR and the downstream fragment was digested by NotI and SacII restriction enzymes and cloned in NAT1 cassette. (TIF) [file ppat.1007460.s002.tif]

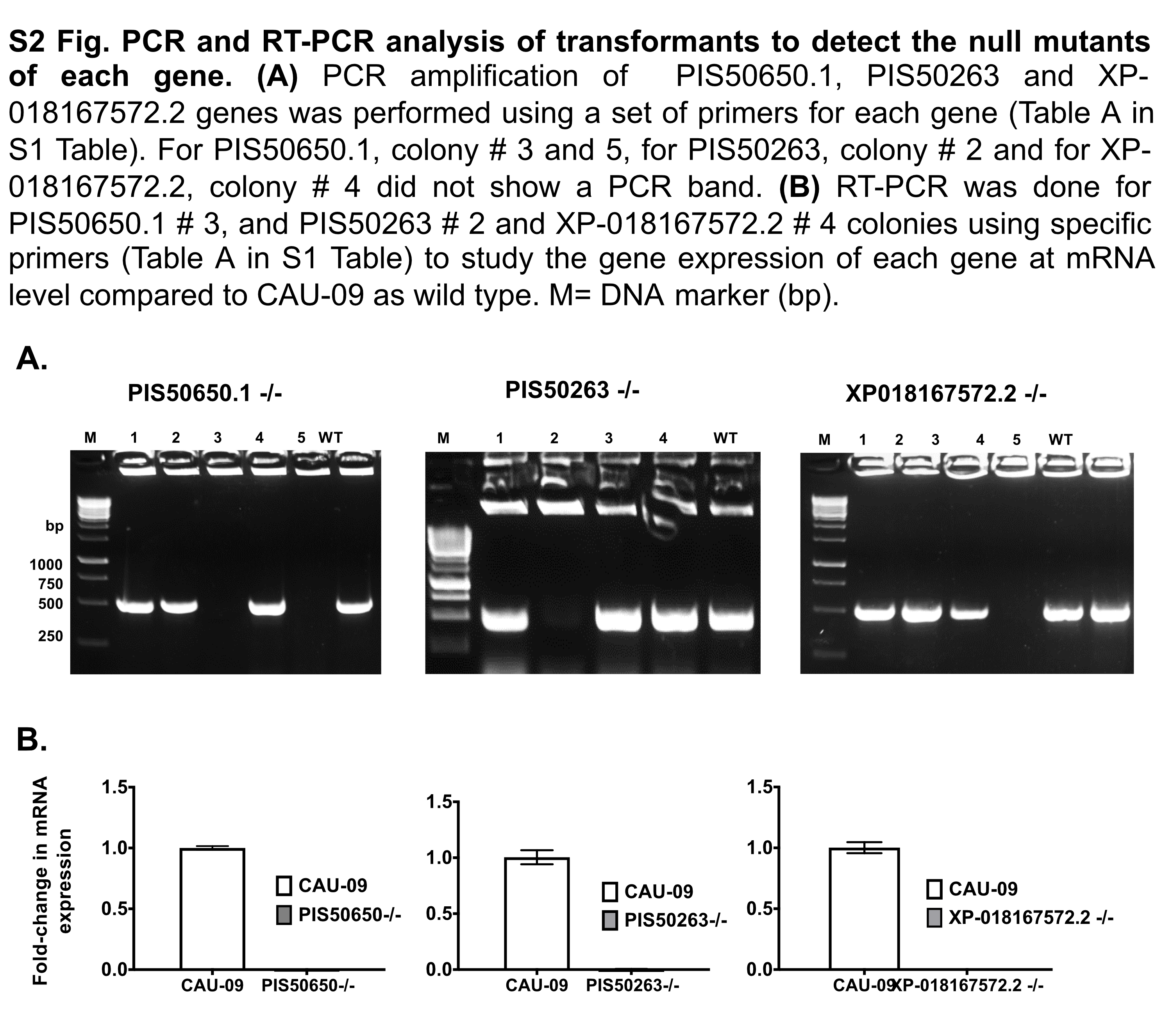

Supplement: S2 Fig — (A) PCR amplification of PIS50650.1, PIS50263 and XP-018167572.2 genes was performed using a set of primers for each gene (Table A in S1 Table). For PIS50650.1, colony # 3 and 5, for PIS50263, colony # 2 and for XP-018167572.2, colony # 4 did not show a PCR band. (B) RT-PCR was done for PIS50650.1 # 3, and PIS50263 # 2 and XP-018167572.2 # 4 colonies using specific primers (Table A in S1 Table) to study the gene expression of each gene at mRNA level compared to CAU-09 as wild type. M = DNA marker (bp). (TIF) [file ppat.1007460.s003.tif]

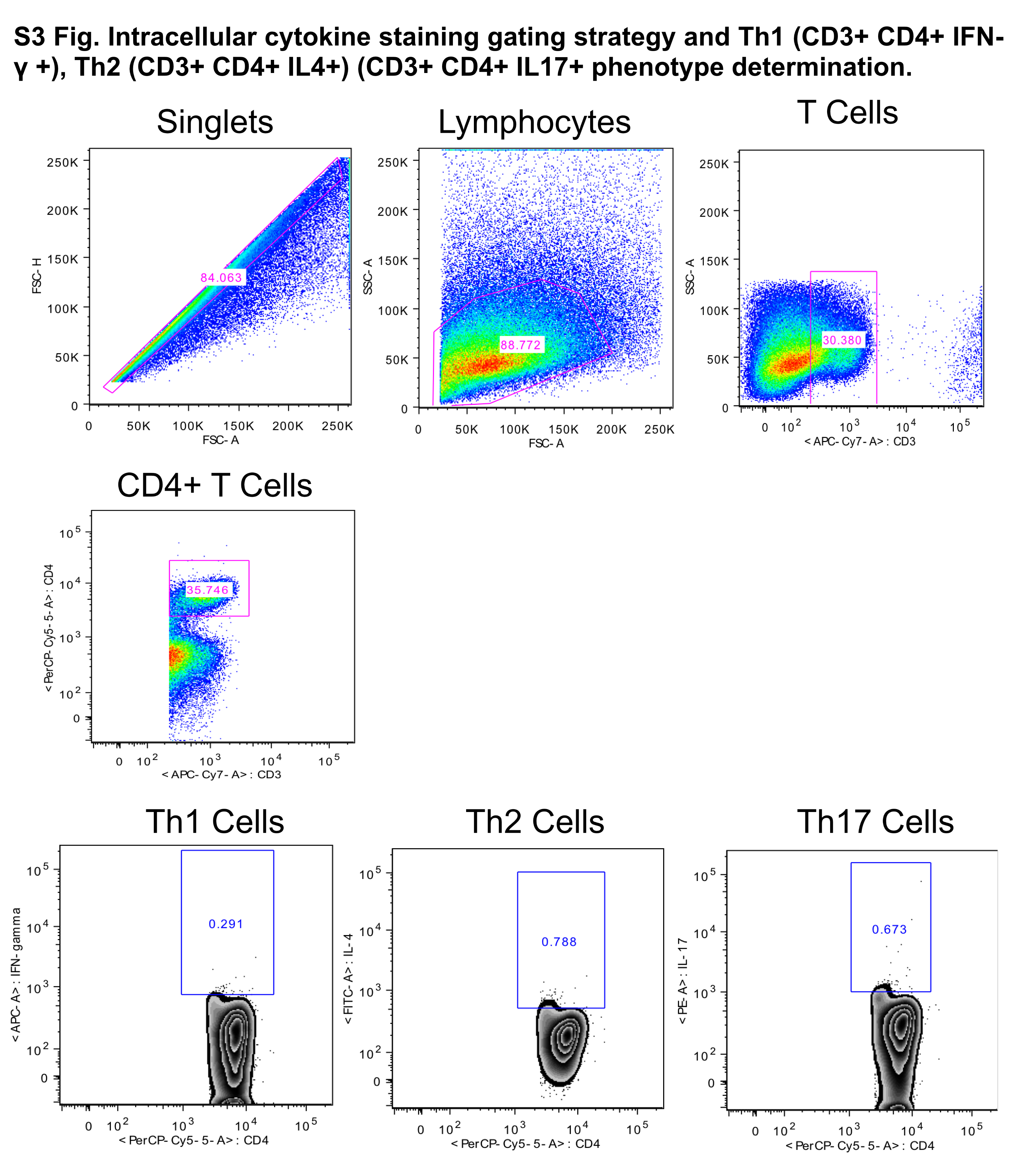

Supplement: S3 Fig — (TIF) [file ppat.1007460.s004.tif]

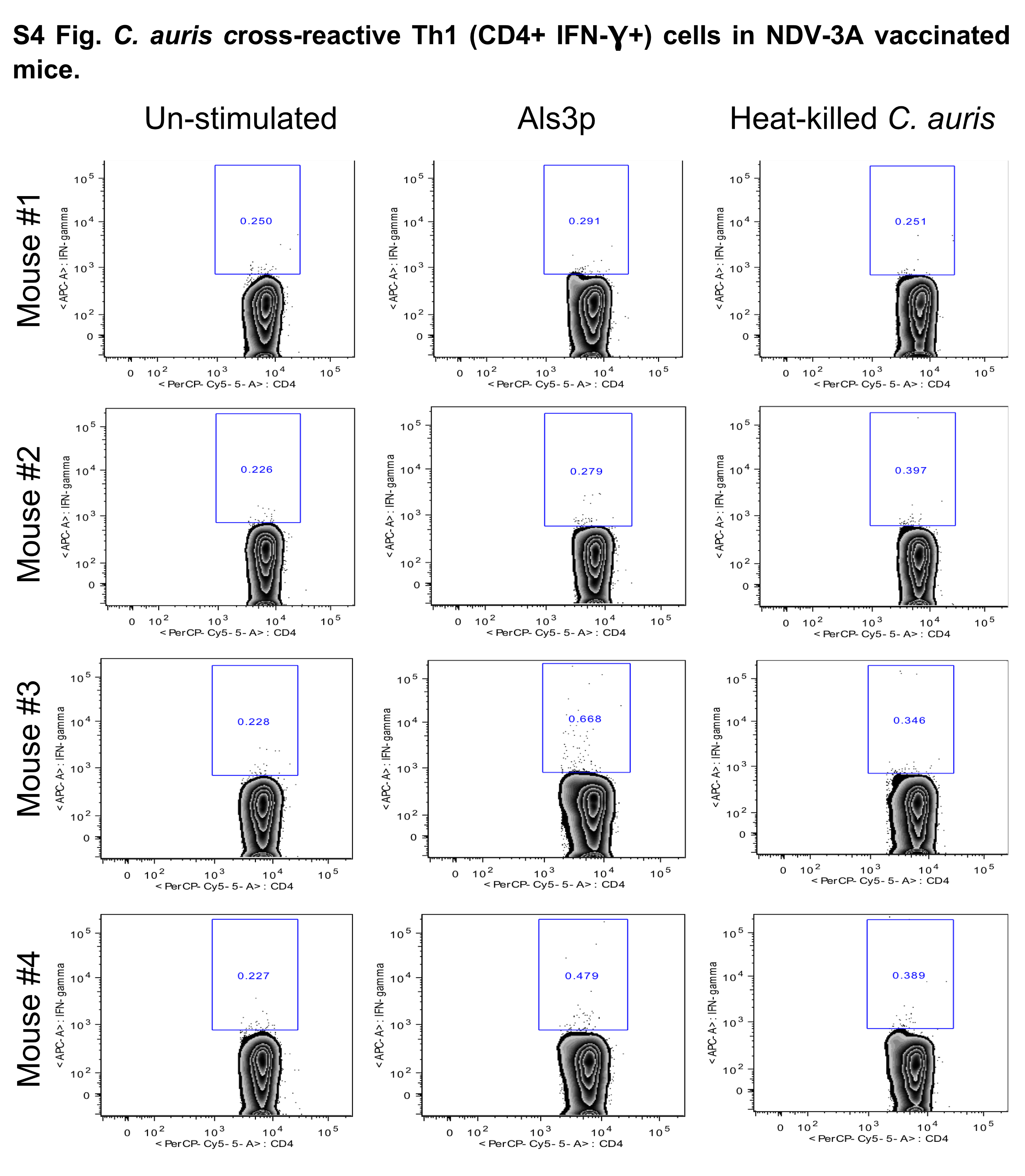

Supplement: S4 Fig — (TIF) [file ppat.1007460.s005.tif]

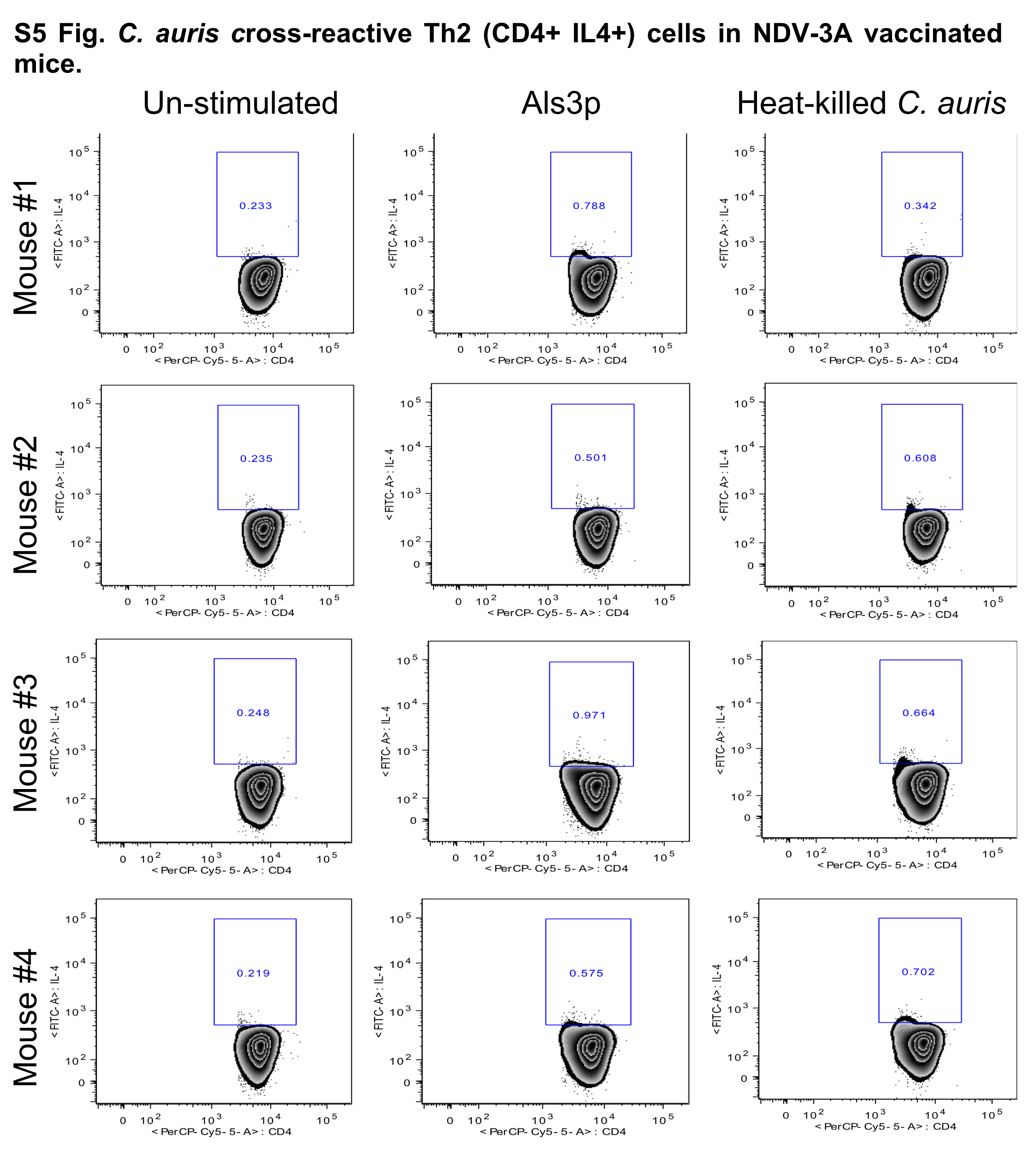

Supplement: S5 Fig — (TIF) [file ppat.1007460.s006.tif]

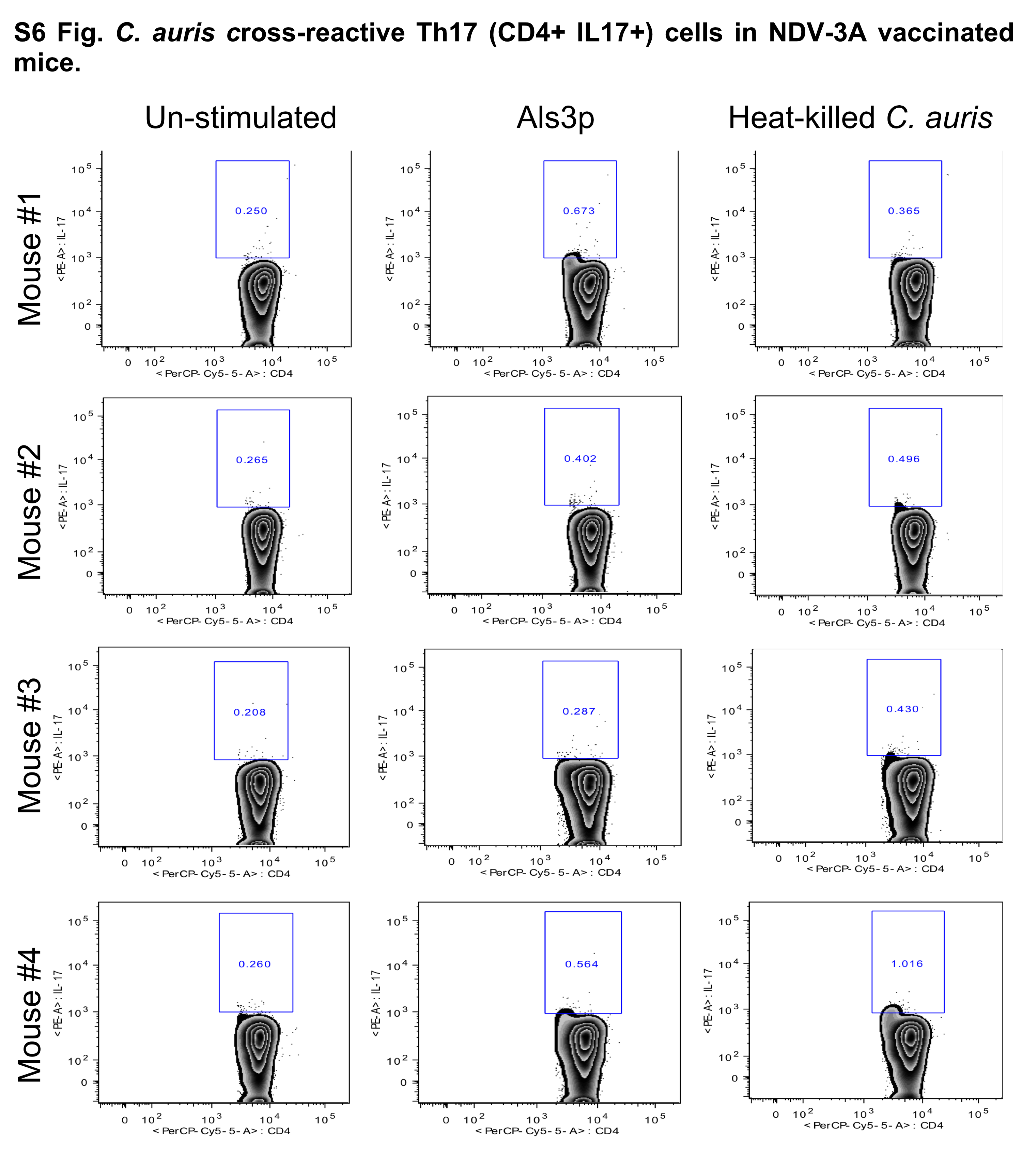

Supplement: S6 Fig — (TIF) [file ppat.1007460.s007.tif]

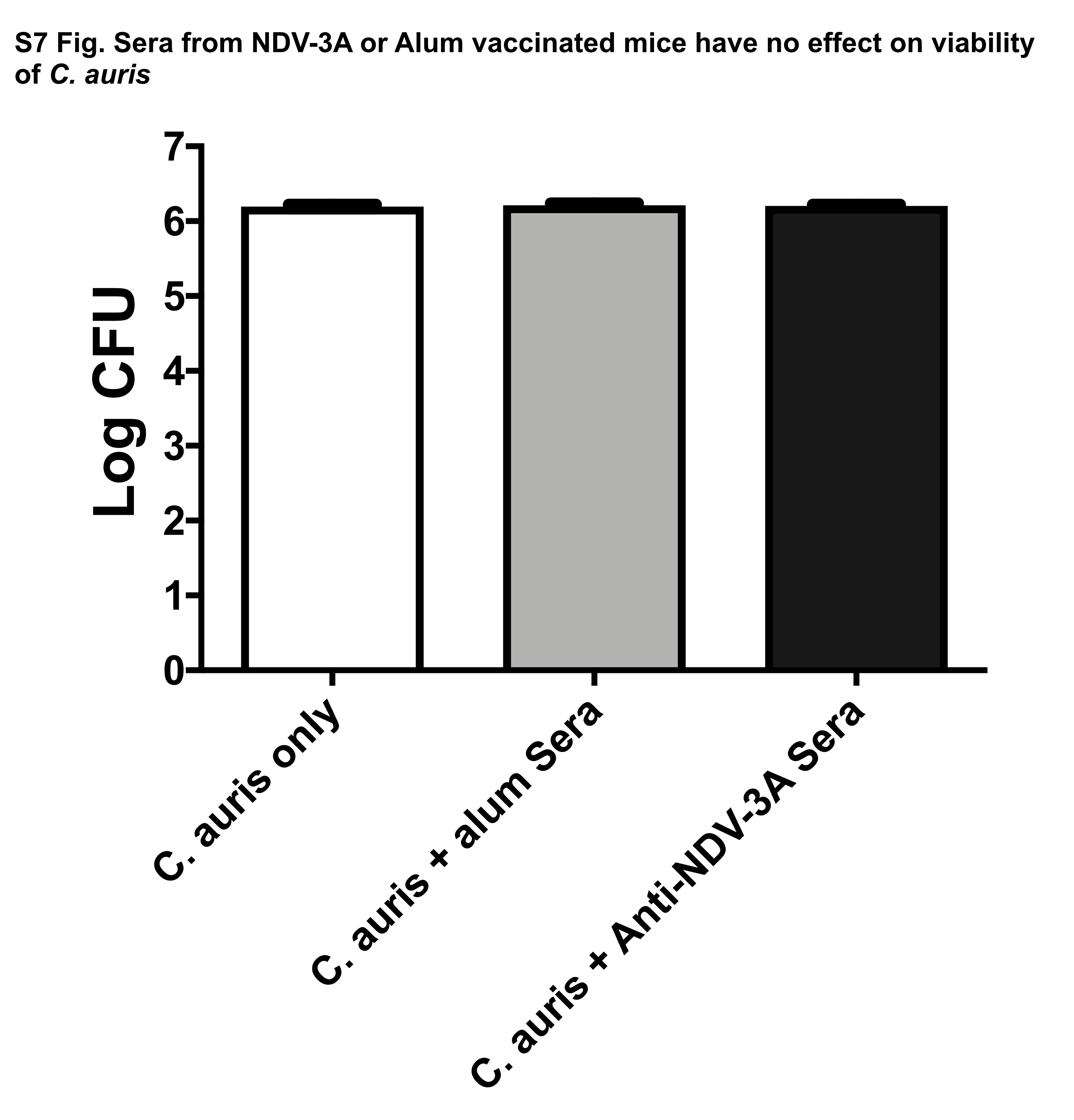

Supplement: S7 Fig — (TIF) [file ppat.1007460.s008.tif]

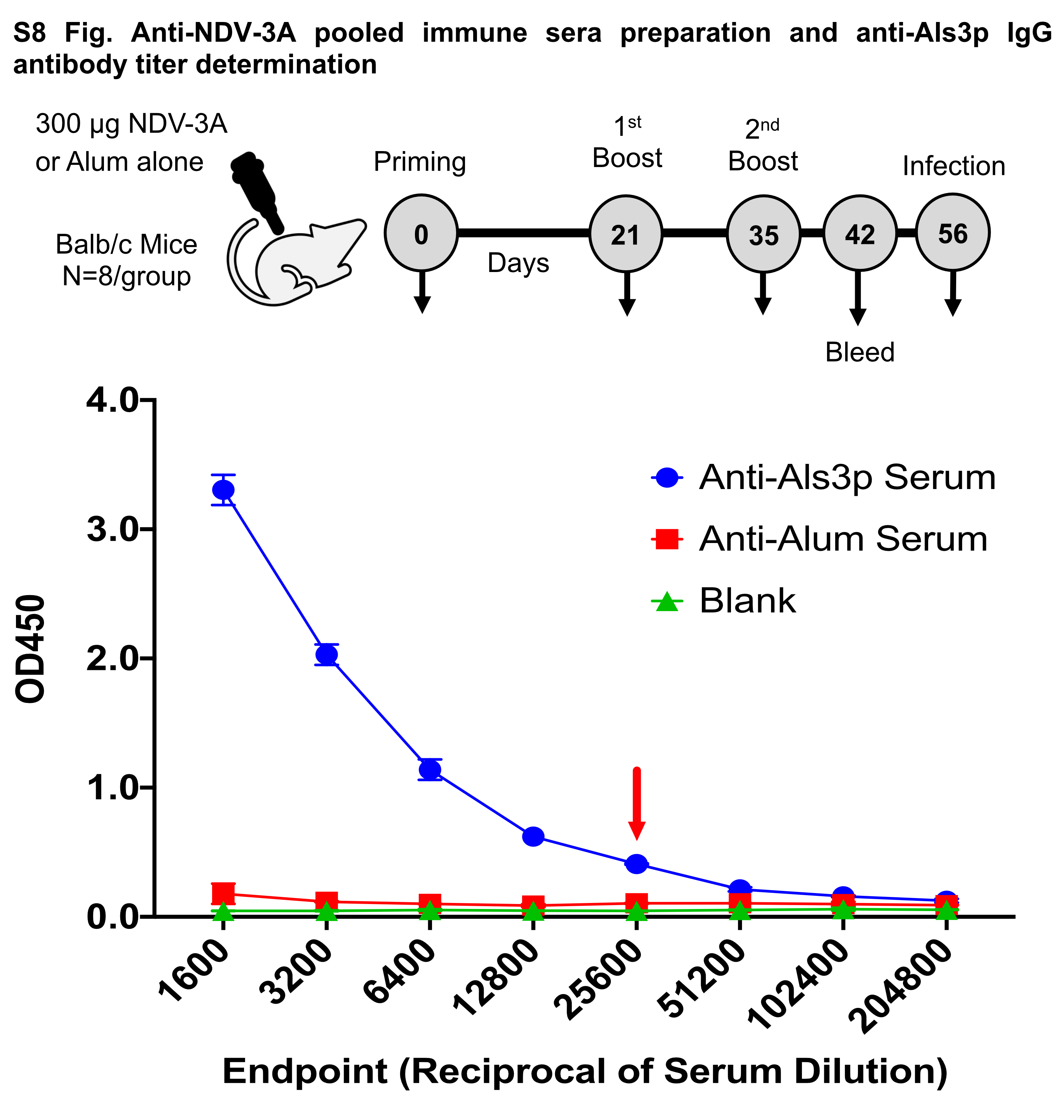

Supplement: S8 Fig — (TIF) [file ppat.1007460.s009.tif]

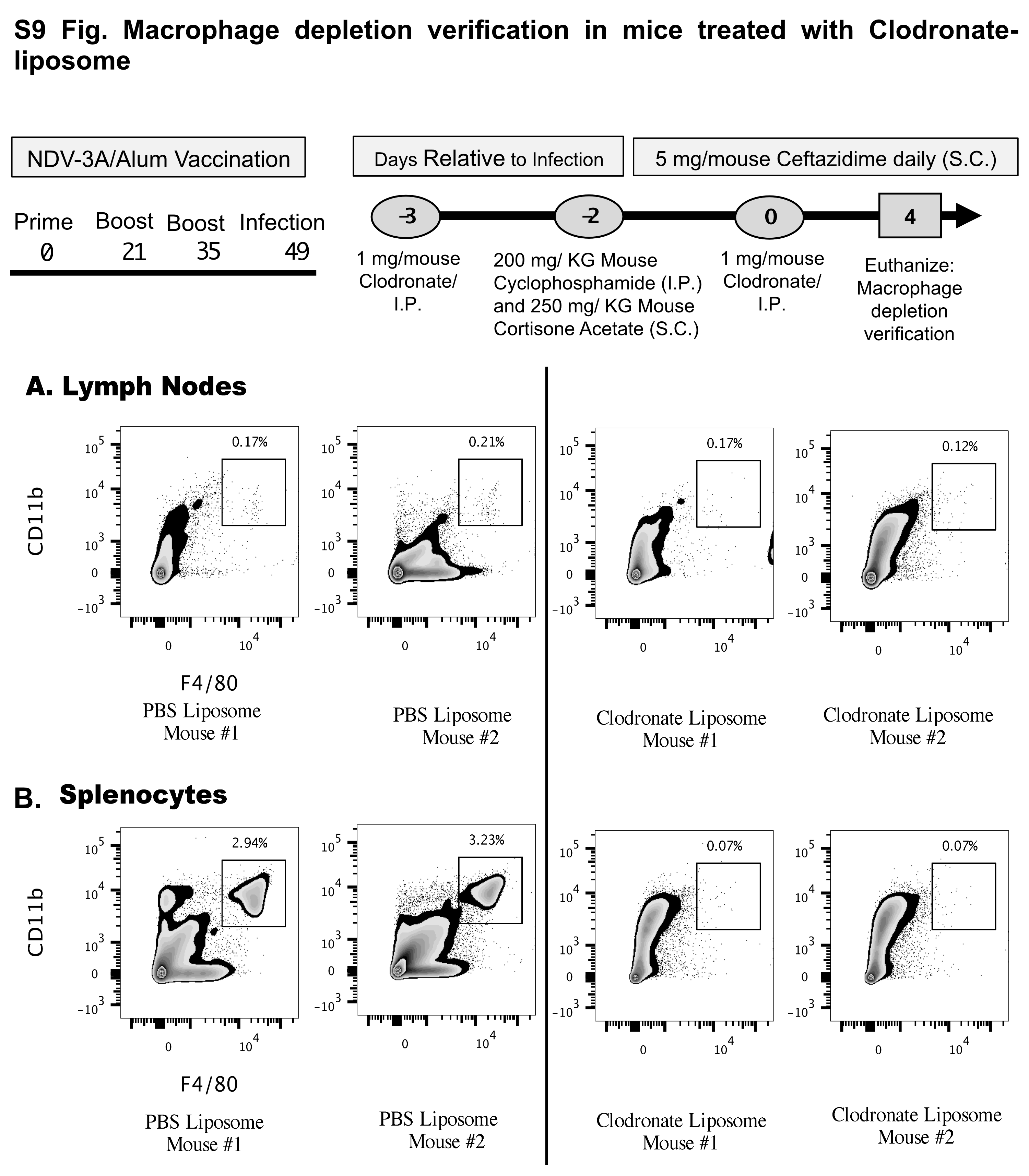

Supplement: S9 Fig — (TIF) [file ppat.1007460.s010.tif]

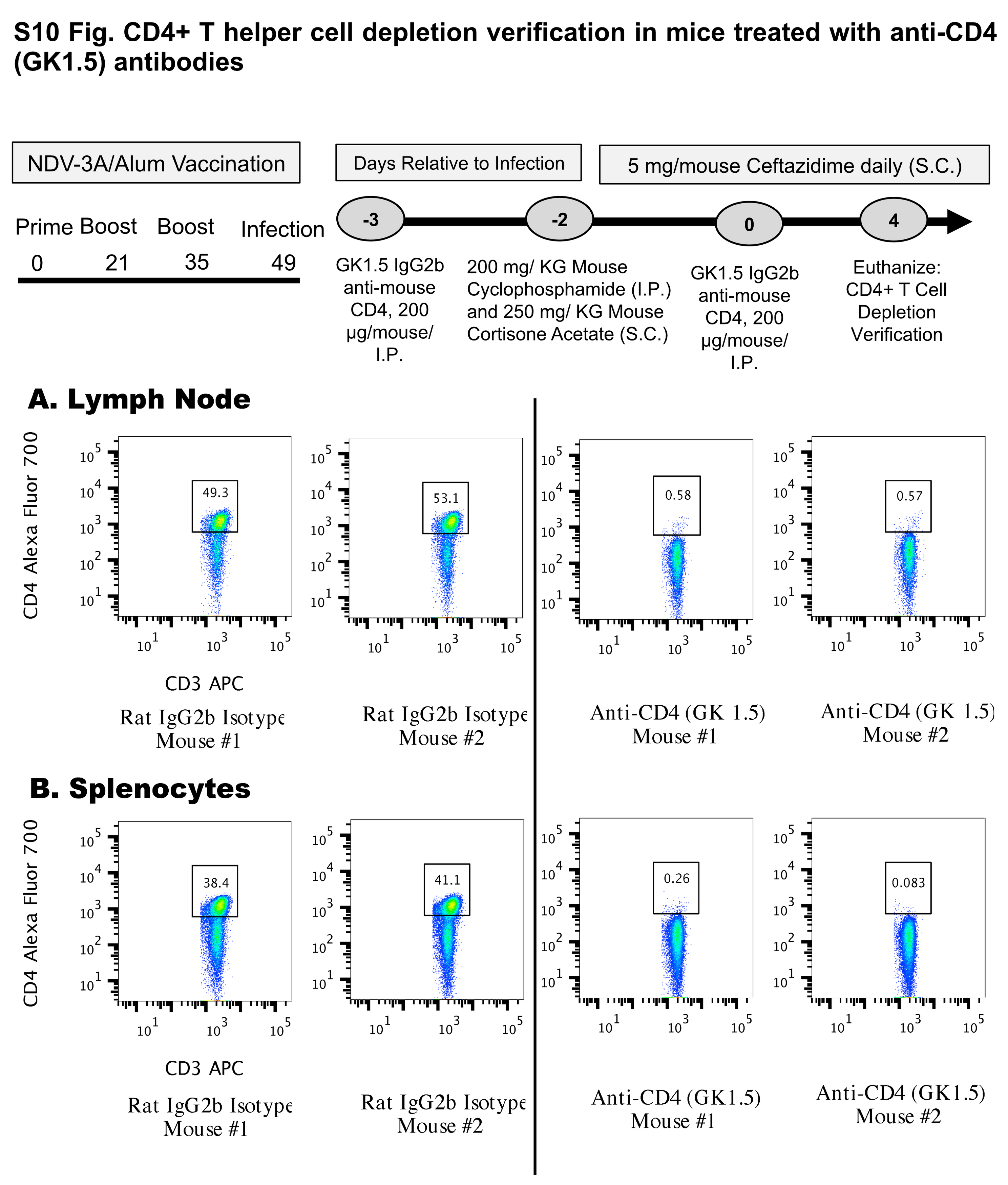

Supplement: S10 Fig — (TIF) [file ppat.1007460.s011.tif]

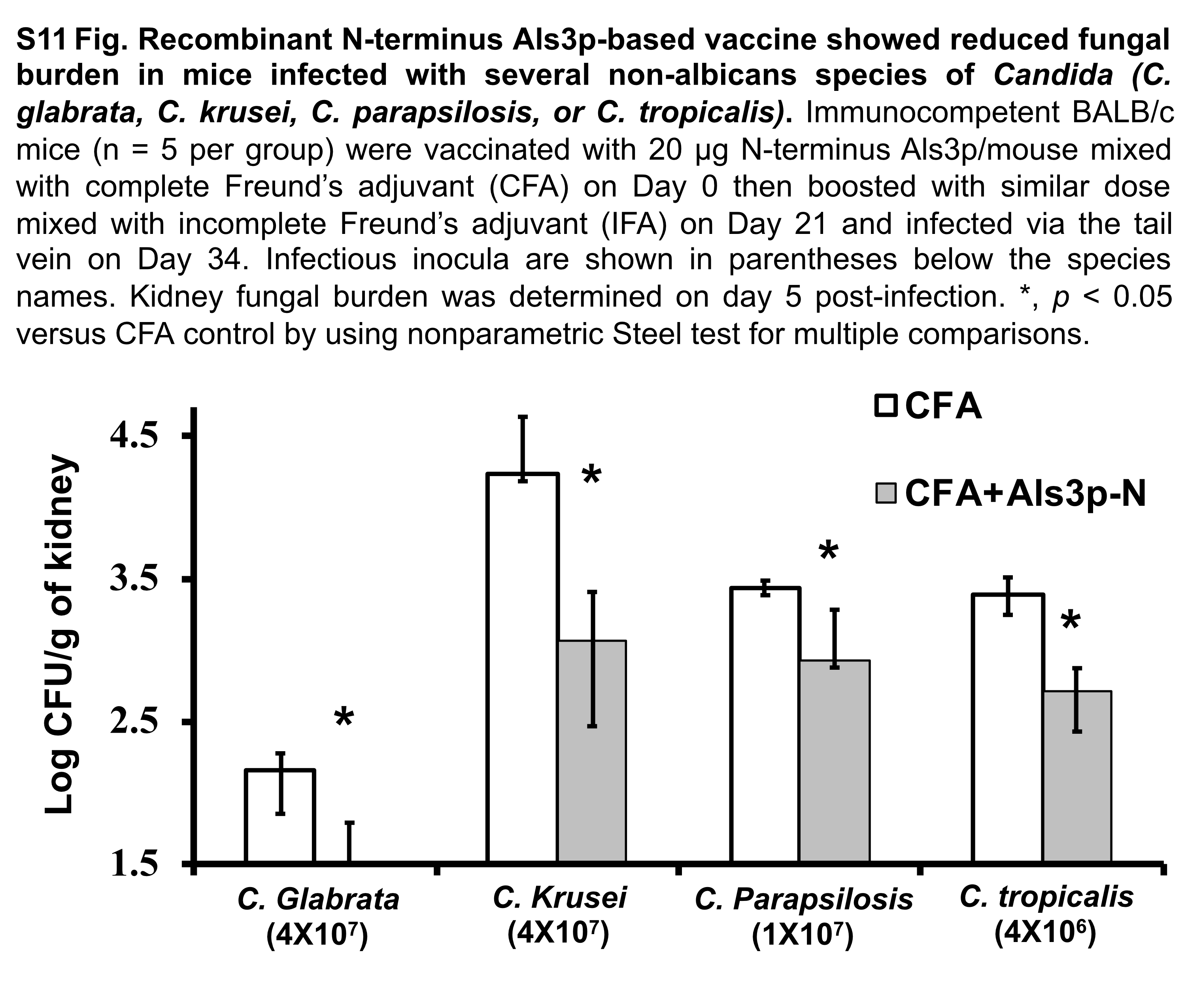

Supplement: S11 Fig — Immunocompetent BALB/c mice (n = 5 per group) were vaccinated with 20 μg N-terminus Als3p/mouse mixed with complete Freund’s adjuvant (CFA) on Day 0 then boosted with similar dose mixed with incomplete Freund’s adjuvant (IFA) on Day 21 and infected via the tail vein on Day 34. Infectious inocula are shown in parentheses below the species names. Kidney fungal burden was determined on day 5 post-infection. *, p < 0.05 versus CFA control by using nonparametric Steel test for multiple comparisons. (TIF) [file ppat.1007460.s012.tif]
