# Supplementary material for: The NDV-3A vaccine protects mice from multidrug resistant Candida auris infection
Source: PLoS Pathog. 2019 Aug 5;15(8):e1007460. doi: 10.1371/journal.ppat.1007460 (PMC6695204; doi:10.1371/journal.ppat.1007460)
Supplement: S1 Table — (A)Primer and guide RNA sequence used in Als3p homolog mutant preparation. (B) MIC of antifungal drugs against different C. auris strains. All MIC are in μg/ml and determined by CLSI method. For azoles and echinocandins, MIC is defined as 50% inhibition, and for amphotericin B as 100% inhibition of different C. auris strains. (DOCX) [file ppat.1007460.s013.docx]

**S1 Table. (A)** **List of primers and guide RNA used in Als3p homolog mutant preparation**

1. Primers used for gene deletion (restriction sites are underlined)

| **Primer** | **Sequence (5′ to 3′)** |
| --- | --- |
| PIS650.1-1F/KpnI | CGG GGT ACC GCT ATC GGT TGA CCC AAT TTG GCC G |
| PIS650-2R/ApaI | GGG GCC CGA AAG ATG ATG GGA AAG AAG GTG AAG G |
| PIS650-11F/NotI | TTG CGG CCG CCT ACA ACT TCC ATT GCC ACA GCC |
| PIS650-12R/SacII | GCC GCG GCA TGG CAT TCG TTC ATA TCC ACA TG |
| PIS50263-G10F | ACT AAA GGG AAC AAA AGC TGG GTA CCA ACA TCC AAC CAA TGT TCT GTG |
| PIS50263-G-2R | GGA ACT TCC TCG AGG GGG GGC CTG ATA AAT GGA AAG TAG AGA AAC ATT AGA CG |
| PIS50263-10F/NotI | TTG CGG CCG CCA AAT GTT GAG TAC CCC TCT GCT |
| PIS50263-11R/SacII | GCC GCG GCT CGG TTG CTC AAA TCA AGG CTA AG |
| XP-018167-12F/KpnI | CGG GGT ACC GAG TAC GTC TAC AAG CGA TAT TAT ATC G |
| XP-018167-2R/ApaI | GGG GCC CGA GCA GTC GAT GCT TCA AAT TAA ATC |
| XP-018167-3F/NotI | TTG CGG CCG CTT CAT CAT ACT GGA GCC AGG ATA TT |
| XP-018167-4R/SacII | G CC GCG GGC ATA CTG AAC GTT TCT TGC ATA TTC |

1. Primers for sequencing

| **Primer** | **Sequence (5′ to 3′)** |
| --- | --- |
| NAT1-1R | TGC TTG CAA AAA TCT AGA ACT AGT |
| NAT1-2F | ACG AGG CAA GCT TGA TGG AAG TTC |
| PIS650-5F | CCT GTG GGT GCT GCT ATT TGC |
| PIS650-8R | CCA CGA CAT GCA AAT TAT CTA CGG AT |
| PIS50263-5F | GAG GGG AGC CTG GTA AAA TC |
| PIS50263-9R | GGA AGA GAA TCG ACA AGA AGT TC |
| XP-018167-5F | GCT ACT TCT CAA TCC CTC TTC TAA CG |
| XP-018167-11R | CGG AGA GAA GCA TTA GAA CCT TAG AT |

1. Primers used for gene deletion verification by PCR

| **Primer** | **Sequence (5′ to 3′)** |
| --- | --- |
| PIS650-9F | CAA CTC CTG TCA CTG TGA TT |
| PIS650-10R | GAA CCA GCA GGT GGC TCA GTG AA |
| PIS50263-6F | GTCAATTCCGCCAAGGGTACC |
| PIS50263-7R | TGAGGAATAGCACTGTCCACATTAAT |
| XP-018167-6F | GATGATACCCTAACTCAAGTTGTAGTG |
| XP-018167-7R | AGTAGATGAATTCATCAGGGACGT |

1. Primers used for gene deletion verification by RT-PCR

| **Primer** | **Sequence (5′ to 3′)** |
| --- | --- |
| Actin-CAU-09-q1F | GTA TGT GCA AGG CCG GTT TC |
| Actin-CAU-09-q2R | TGG ATT GAG CCT CAT CAC CG |
| PIS50650-q1F | CTA CAA CCA AAA GCG GCA CC |
| PIS50650-q2R | GTT TCT GTG GTG GAT CCC GT |
| PIS50263-q1F | TGA GAA AGG CCG TTA TGC GT |
| PIS50263-q2R | CAA CGA CCG ACA GAC AGT CA |
| XP-01816-q1F | CGC CAC AGT ATC GTG GCA TA |
| XP-01816-q2R | TGG TGC CGT CAC TCA TTT GT |

1. Guide RNA (gRNA) sequence (PAM “ NGG”)

| **Gene** | **Guide RNA Sequence** |
| --- | --- |
| PIS650.1 | GTTCTGGTGACGGTGGTCGT**AGG** |
| PIS50263 | ATGGACCCAAGACAAGGTGC**TGG** |
| XP018167572.2 | GTTCCACCAACGAAGGTAAT**CGG** |

**S Table.** **(B)** **MIC of antifungal drugs against different *C. auris* strains**

| Strain | Clade | FZ | VZ | IZ | PZ | AF | CF | MF | 5-FC | Amp B |
| --- | --- | --- | --- | --- | --- | --- | --- | --- | --- | --- |
| CAU-01 | II | 4 | 0.03 | 0.125 | 0.06 | 0.25 | 0.125 | 0.125 | 2 | 0.38 |
| CAU-03 | III | 128 | 4 | 0.5 | 0.5 | 1 | 0.25 | 1 | 0.5 | 0.38 |
| CAU-05 | IV | >256 | 16 | 1 | 1 | 1 | 0.5 | 0.5 | 0.5 | 0.5 |
| CAU-07 | I | 8 | 0.6 | 0.5 | 0.25 | 0.5 | 0.25 | 0.5 | 8 | 0.75 |
| CAU-09 | I | 256 | 4 | 0.25 | 0.125 | 1 | 0.5 | 0.25 | 128 | 4 |

All MIC are in μg/ml and determined by CLSI method. For azoles and echinocandins, MIC is defined as 50% inhibition, and for amphotericin B as 100% inhibition of different *C. auris* strains.

**Abbreviations:** Minimal Inhibitory Concentration (MIC), Fluconazole (FZ), Voriconazole (VZ), Itraconazole (IZ), Posaconazole (PZ), Anidulafungin (AF), Caspofungin (CF), Micafungin (MF), 5-Flucytosine (5-FC) and Amphotericin B (Amp B).
